# Seismic Safety Design and Analysis of Hydraulic Sluice Chamber Structure Based on Finite Element Method

**DOI:** 10.1155/2022/6183588

**Published:** 2022-09-16

**Authors:** Xin Cai, Zhenming Cui, Xingwen Guo, Fan Li, Yanan Zhang

**Affiliations:** ^1^College of Water Conservancy and Hydropower Engineering, Hohai University, Nanjing 210000, China; ^2^College of Mechanics and Materials, Hohai University, Nanjing 210000, China

## Abstract

One of the important links in the safety evaluation of sluices is the aseismic safety examination. In order to ensure the daily safe operation of sluices, it is necessary to conduct a normalized aseismic safety examination of sluices, and it is also necessary to study the aseismic safety examination of return sluices. Based on the application of ADINA finite element analysis software, a three-dimensional finite element model of the gate chamber structure is established, and the seismic response of the gate chamber structure is calculated and analyzed by the mode decomposition response spectrum method. The seismic safety of the gate chamber structure is evaluated comprehensively. The results show that 2.00 MPa of tension stress is generated at the junction of the pier and the gate. According to the structural mechanical method, the maximum tensile stress that can be endured is 4.41 MPa, which meets the safety requirements. There is a large tension stress zone between the elevator floor and some parts of the elevator, which far exceeds the standard tension strength value of the concrete moving shaft. Considering the safety, corresponding aseismic reinforcement measures should be taken. The structure of the gate chamber is nonslip and stable, and the safety factor is larger than the standard value of the Gate Design Specification (SL265-2016), which meets the safety requirements. The aseismic safety of the gate chamber structure meets the requirements of the “Standard for Seismic Design of Water Conservancy Buildings” (GB5127-2018), but it has safety defects and the aseismic grade is *B*.

## 1. Introduction

According to the national water conservancy safety data in 2009 and the record data of sluice in 2008, there are 97 small (1) type or above directly managed sluice in the Yellow River Basin. The main structural forms are divided into open type and culvert type [1]. The basic stratum types of the gate are generally the fourth system of the altered series, the upper altered series of the fourth system, the middle altered series of the fourth system, the lower altered series of the fourth system, the upper Triassic series, and the upper Cambrian series, which are mainly caused by artificial accumulation, alluvial deposition, and flood. The soil properties are divided into 24 different lithology, such as fine sandy loam, loam, sandy soil, sandy soil, sandy gravel, light silty loam, sandy soil, silty sand, and screened fine soil. The second largest earthquake area in China's history is the North China earthquake zone [2].

Due to the change of the seismic design specifications of domestic buildings, the seismic design of locks mainly faces the following three problems: (1) the original engineering design did not fully consider the seismic protection and the seismic protection needs to be fully considered according to the standard. According to the existing national seismic work parameter zoning map (gb18306-2015), the structure of building fortification strength and seismic capacity assessment needs to be redefined; (2) the original design considered earthquake protection, but the building fortification strength is low, such as 6 and 7 degrees. Now, according to the code for seismic design of water conservancy building materials (sl191-2008), the fortification strength has increased, and the seismic capacity needs to be reviewed; (3) earthquake is considered in the original design, and the fortification intensity has not changed [3]. Strengthening the protection measures against earthquakes leads to insufficient earthquake resistance in the original design, and it is necessary to focus on the review of the protection measures against earthquakes [4]. Today, with the development of calculation methods and the improvement of social and economic technology, the seismic characteristics of building materials will become increasingly important. Compared with the current requirements for seismic fortification, the initial design of gate using quasi-static calculation method is slightly insufficient. Therefore, more advanced calculation methods and theories are needed to analyze the comprehensive seismic capacity of buildings.

There are many natural disasters caused by earthquakes and they occur relatively frequently in China. Seismic safety evaluation is the main part of ship lock safety evaluation project. The throat of ship lock construction is the lock chamber, and the design of the lock chamber plays an important role in the safety of the project. Therefore, it is necessary to discuss the seismic stability of the gate chamber structure. At present, most researchers use quasi-static method to review and analyze the gate structure [5]. In fact, the sluice is a three-dimensional thin-wall structure [6]. Connections between buildings, such as traffic bridges, have large errors in the results [7]. In recent years, with the rapid development of computer technology, the finite element numerical simulation technology has been widely used in the seismic analysis of sluices [8]. Therefore, it is necessary to use the three-dimensional finite element numerical simulation technology to study the seismic resistance of the gate chamber structure [9].

## 2. State of the Art

### 2.1. Overview of Sluice Gate

The gate refers to a low head hydraulic structure that can adjust the flow of the water body by opening the gate to adjust the flow or adjust the temperature. As the gate plays a major role in regulating water flow and generating electricity, it is widely used in the field of water conservancy. As early as the spring and autumn period, the Chinese began to build ship locks. It is reported that the earliest ship lock in China was built in today's Anhui Province. There are five ship locks in China. According to statistics, by 2008, China had built more than 50000 flood discharge gates, including more than 480 large-scale flood discharge gates, which improved the local drainage and irrigation capacity and accumulated rich experience in promoting projects.

#### 2.1.1. Classification of Sluice Gates

According to the different responsibilities of sluice structures in water conservancy, sluice gates can be classified as follows:

1. *Control the brake*. Generally speaking, the control brake is built on the barrage between the main river and the branch river, and the water level is controlled through the ship lock to meet the requirements of upstream and downstream water diversion and navigation, control the upstream and downstream water flow to ensure the safety of the upstream and downstream rivers, or control the flow direction according to the downstream water supply requirements [10]. If the control gate and the sluice are built together somewhere, it can not only control the inflow flow but also completely discharge the excess water, thus protecting the safety of hydraulic structures [11].
2. *Entry lock* (*entrance lock*). It is mostly built on the banks of rivers, ponds, or lakes or above the diversion channels of agricultural irrigation areas to regulate the amount of tap water and ensure the demand of agricultural irrigation water supply, industrial water, and domestic water [12].
3. *Flood gate*. Generally, mountain torrents, which are mostly located on the side of the river and larger than the safe flow of the downstream river, can be divided into flood storage area, flood detention area, and flood diversion channel [13].

#### 2.1.2. Components of the Sluice

According to the composition of sluice, it can be generally divided into three parts: (1) sluice chamber [14]and (2) upstream connection section [15]. The sluice chamber is the most important part in the structure of the sluice. The sluice chamber consists of the bottom plate, middle pier, side pier, gate, daughter wall, maintenance bridge, work bridge, and traffic bridge. The upstream and downstream water level and flow can be changed by adjusting the opening of the gate. The bottom plate of the gate can reduce the effect of osmotic pressure on the whole gate. All the structures of the room are self-respecting. Upstream connection section is usually composed of the above subgrade, bottom protection, upper inverted chute, wing beam, and embankment on both banks. In particular, the mat ensures the chamber and reduces scour and osmotic pressure. The upstream bottom and the squeezed riverbed greatly reduce the scouring of the water flow. The upstream wing wall is designed to form a favorable water flow situation, let the water flow smoothly into the gate area, and achieve the functions of soil blocking, anti-scouring, and anti-lateral seepage.

### 2.2. Calculation Theory of Sluice Seismic Design

#### 2.2.1. Influence of Earthquake on Sluice Gate

**Figure 1:**
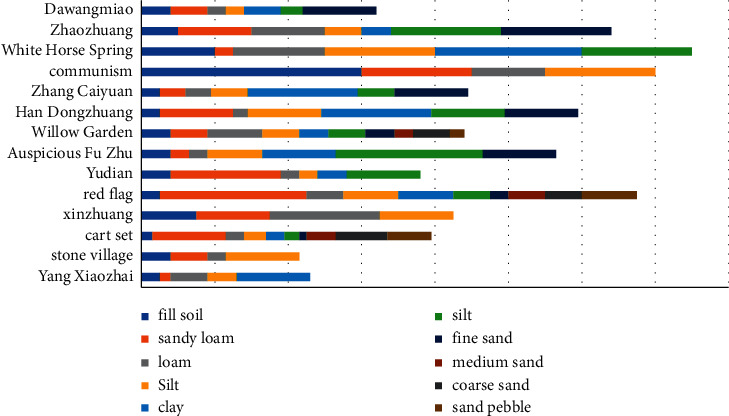
Geological column map of the bottom floor of the sluice gates in the middle and lower reaches of the yellow river.

The gate structure has been damaged by strong earthquakes for many times, which seriously affected the normal operation of the gate. However, according to the statistics of the earthquake disaster data after the previous major earthquakes, the main earthquake disaster causes of the gate in China are the fracture of the protection pool, the bottom plate, the damping pool, and the bottom plate structure. The main factors leading to this fracture phenomenon are as follows: (1) the strong earthquake resistance causes the imbalance of the sluice, causing structural displacement, fracture, and even collapse or uplift; (2) under the influence of earthquake damage force, the strength and safety of the structure are damaged, resulting in fracture, deflection, and even collapse [2], and due to the influence of earthquake force, the strength and safety of the structure are damaged, resulting in cracks, deflection, and even collapse [16]. Earthquake resistance is a complex and changeable natural event. There are many factors that affect the impact of earthquake on the gate. The main reasons are as follows: (1) seismic speed and time; (2) the shockproof function of the gate itself; (3) the basic geological structure of the gate, the time of its generation, and the function of the building; and (4) static load applied on the sluice structure [17]. According to the Geological Survey Report of Locks in the Middle and Lower Reaches of the Yellow River and the Engineering Geological Research Atlas of the Lower Reaches of the Yellow River, 42 locks in the middle and lower reaches of the Yellow River are investigated. And the histogram of their foundation soil quality is shown in Figure 1.

**Figure 2:**
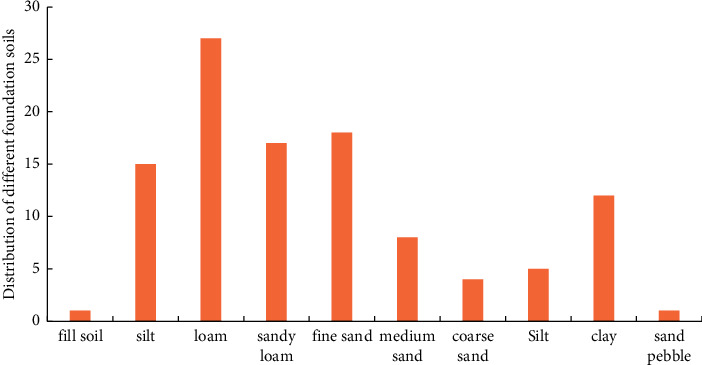
The composition of different foundation soils of the bottom plate of the sluice gate in the middle and lower reaches of the yellow river.

According to the geological conditions, soil types and soil thickness of 42 sluice gates in the middle and lower reaches of the Yellow River, the average thickness of various soil layers is calculated. The calculation results are as follows: the proportion of fill is 0.5%, the proportion of silt is 15.1%, the proportion of loam is 26.3%, the proportion of fine sand loam is 17.1%, the proportion of screened fine soil is 17.5%, and the proportion of neutral clay is 7.3%. The proportion of coarse sand is 2.2%, the proportion of silt is 2.8%, and the proportion of clay is 10.7%, and Figure 2 shows the composition map of the foundation soil of the sluice chamber section in the middle and lower reaches of the Yellow River.

## 3. Methodology

### 3.1. Seismic Calculation Theory of Sluice Gate

#### 3.1.1. Seismic Response of a Single Degree of Freedom System

The so-called single degree of freedom system means that the structure is only a motion acceleration system [18]. In the seismic analysis, the ground is assumed to be in rigid motion, and the structure is a vibration system with mass *M*-spring *K*-damping *c* located on this rigid plane. According to Newton's second law, the following relationship can be obtained:

(1) $$\begin{array}{c} M\ddot{x}\left(t\right)+c\dot{x}+Kx\left(t\right)=-M{\ddot{x}}_{g}\left(t\right)\text{.} \end{array}$$

In construction engineering, the fixed vibration period *T* of the structure is generally used as the fixed vibration frequency *ω* (*t* = 2*π*/a) to represent the seismic response spectrum [19]. There are the following approximate relationships between relative displacement, relative velocity, and absolute acceleration:

(2) $$\begin{array}{c} S_{D}\left(\omega ,\xi \right)={\left|x\right|}_{\max}=\frac{S_{V}\left(\omega ,\xi \right)}{\omega }\text{,} \end{array}$$

(3) $$\begin{array}{c} S_{A}\left(\omega ,\xi \right)={\left|\ddot{x}+{\ddot{x}}_{g}\right|}_{\max}=\omega^{2}S_{D}\left(\omega ,\xi \right)\text{.} \end{array}$$

Because of these relationships, some textbooks often draw three kinds of spectra together, which is also called three coordinate spectrum, or three spectrum diagram [19]. Seismic acceleration response spectrum is very common in building seismic engineering.

#### 3.1.2. Seismic Response of Multi-DOF Systems

Finite element method is the most commonly used method to analyze seismic response of multi-degree-of-freedom systems. The array superposition method is commonly used in seismic response analysis of sluice. The first step of the analysis and research is to deal with the characteristics of the structural system so as to achieve the first few low-order structures with large research on the vibration of the structural system. The second step is to calculate the motion equation of the system through the orthogonality of the stratum. Using the decoupling method, the stratum responses corresponding to different strata are obtained, and then, the responses of each stratum are superposed according to certain principles to obtain the comprehensive seismic response of the structure. The free vibration equation of the undamped multi-degree-of-freedom linear system after finite element discretization is

(4) $$\begin{array}{c} \left[M\right]\left\{\ddot{u}\left(t\right)\right\}+\left[K\right]\left\{u\left(t\right)\right\}=\left\{0\right\}\text{,} \end{array}$$

where [*M*] and [*K*] are the overall stiffness matrix and mass matrix of the system, respectively; {*u* (*t*)} is the displacement reflection of the system.

(5) $$\begin{array}{c} \left\{u\left(t\right)\right\}can\;be\;expresse\;d\;as:\left\{u\left(t\right)\right\}=\left\{U\right\}\sin\left(\omega t+\phi \right)\text{.} \end{array}$$

Since the formation of the system has the characteristic of weighted orthogonality, its dynamic response can be expanded according to its formation, that is,

(6) $$\begin{array}{c} \left\{u\left(t\right)\right\}=\overset{N}{\underset{i=1}{\sum }}q_{j}\left(t\right)\text{,} \end{array}$$

where *q*_*j*_ (*t*) is the generalized coordinate of the amplitude change of the formation, which reflects the contribution of the *j*th-order formation to the total response of the system at time *t*.

Modal superposition method can get the response characteristics of the structural system in the whole seismic response, so it is called modal superposition time history analysis method. In the design practice, usually, the maximum seismic response of the system is the most unfavorable and most valued. Therefore, on the basis of modal superposition time history method, a new superposition response spectrum method is formed by combining the theory of seismic response spectrum and modal superposition theory so that the maximum seismic response of the system can be calculated simply. At present, this technology has been applied in the seismic engineering design of building engineering.

The focus of the mode shape superposition response spectrum method is to find the maximum value of the structural response, so it is necessary to first obtain the maximum value of the structural response of each mode shape. It can be seen from the above formula that the maximum value {*S*} *j* of the relative displacement response corresponding to the mode shape *j* can be expressed as

(7) $$\begin{array}{c} {\left\{S\right\}}_{j}={\left\{u\left(t\right)\right\}}_{j}^{\max}=\gamma_{j}{\left\{\Phi \right\}}_{j}{\left|\delta_{j}\left(t\right)\right|}_{\max}\text{.} \end{array}$$

#### 3.1.3. Mode Shape Decomposition Response Spectrum Method

**Figure 3:**
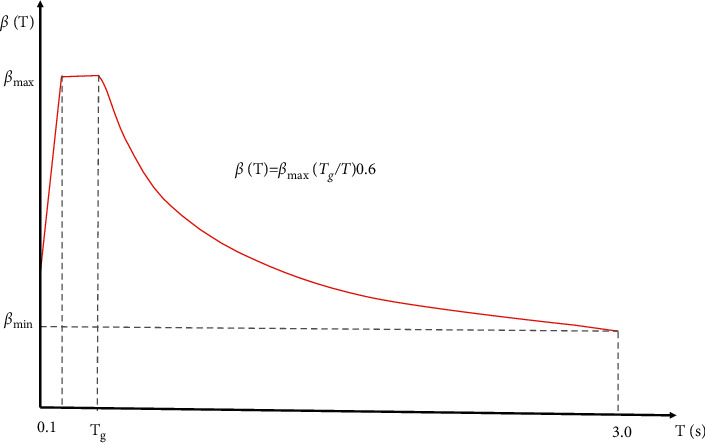
Standard design response spectra.

This time, the standard design maximum response spectrum specified in the “standard for seismic design of hydraulic structures” (cb51247-2018) (hereinafter referred to as the design code standard) will be used for the seismic evaluation and design of the gate chamber design. As shown in Figure 3, the damping ratio is about 7%, and the representative value of the maximum response spectrum *β* and Max is 2.25.

According to the provisions of the national standard value of earthquake resistant design strength, when the seismic action effect is estimated by using the mode decomposition response spectrum method, the seismic action effect of each mode can be combined according to the square root and square root. The specific calculation formula is as follows:

(8) $$\begin{array}{c} S_{E}=\sqrt{\overset{m}{\underset{i}{\sum }}\overset{m}{\underset{j}{\sum }}\rho_{ijS_{i}S_{j}},} \end{array}$$

where *S*_*E*_ is the seismic action effect; *S*_*i*_ and *S*_*j*_ are the seismic action effect of the *i*th and *j*th order modes, respectively; *m* is the number of modes used in the calculation.

### 3.2. Tensile Stress Review

At present, there is very little information about how to evaluate the stability in the range where the tensile stress is greater than the standard deviation of the axial tensile strength of concrete in the finite element calculation. However, according to the conclusion of the finite element seismic review of the open gate, it is not difficult to see that in the case of a large earthquake, the intersection between the gate pier and the gate bottom plate can often produce a large tensile stress area, and the tensile stress in these areas is often greater than the standard value of the concrete dynamic axial tensile strength. Under the influence of earthquake, the gate pier is an eccentric compression member. Therefore, for the sake of safety and considering the worst case, the gate pier can be considered as a pure bending structure. According to the code for design of hydraulic concrete structures (sl191-2008), considering the bending capacity of the positive diameter and the normal rectangular section or inverted *T*-shaped section of the flange at the tension side, the bending capacity of the positive diameter of the bending member must meet the following conditions:

(9) $$\begin{array}{c} KM_{s}⩽f_{c}bx\left(h_{0}-\frac{x}{2}\right)+f_{y}^{\prime }A_{S}^{\prime }\left(h_{0}-a_{s}^{\prime }\right)\text{.} \end{array}$$

In the formula: *K* is the safety factor of bearing capacity; *M*_*s*_ is the design value of bending moment, N.m; *f*_*c*_ is the design value of concrete axial compressive strength, Pa; *A*_*s*_ is the cross-sectional area of longitudinal tension steel bar, m^2^.

Meanwhile, for pure curved surface components, the normal stress at any point on the section can be measured. The equation is

(10) $$\begin{array}{c} \sigma =\frac{My}{I_{z}}\text{,} \end{array}$$

where *m* is the maximum bending moment on the longitudinal section. To obtain the maximum normal stress acceptable on the longitudinal section, here *M* can be taken as *MS*, and *I* is the maximum moment of inertia on the longitudinal section with respect to the neutral axis *Z*; *Y* is the required maximum internal stress.

By comparing the stress data obtained by the above method with the finite element results, the range where the tensile stress is greater than the standard value of the dynamic axial tensile strength of cement can be checked.

## 4. Result Analysis and Discussion

### 4.1. Finite Element Model Construction and Parameter Design

There are four holes in a gate project, i.e., two reinforced concrete open sluice gates, with joints between each joint. The total length of the gate chamber is 12.50 m, the total width of the gate chamber is 47.45 m, the thickness of the side pier is 1.40 m, the thickness of the middle pier is 1.40 m, the thickness of the middle joint pier is 1.85 m, and the net width of each hole is 10.00 m. Considering the difference of earth pressure and water pressure at the two ends of the side pier, this calculation will focus on the combination of the side hole and the gate chamber.

According to the specific standards of the gate chamber design, the structure set includes the gate bottom plate, gate pier, steel gate, cross beam, and side hole 3D finite element model of the hoist frame structure. Using Cartesian coordinate system, take *x* as horizontal azimuth, *y* as downstream azimuth, and *Z* as vertical azimuth. In the calculation process, three-dimensional stability constraints are applied to the bottom of the gate pier and gate. However, it must be noted that the construction of the hub has been carried out for more than 50 years. According to the monitoring data, the land subsidence has been basically balanced. The maximum displacement value is less than 0.5 mm. Therefore, for the convenience of analysis and calculation, the influence of foundation is ignored. In addition, since the gate system of the pulling machine room on each pier of Luqiao road exists separately, thin-layer elements are set between the two adjacent road and bridge construction and hoisting engineering rooms for finite element calculation. The relative independence between the two lifting bays and the highway bridge can be realized without participating in the calculation.

In this calculation result, if the reinforcement unit is not considered, any cement unit represents plain cement. In order to reflect the effect of reinforcement diameter on the elastic modulus of cement, the equivalent elastic modulus is used to simulate the elastic modulus of reinforced concrete. These calculation results of the material parameters used are listed in Table 1.

### 4.2. Experimental Results and Analysis

The structure's self-vibration characteristics are analyzed by means of structure's self-vibration characteristics. Considering the influence of water in front of the gate on the structure of the gate chamber and the self-vibration of the first five frequencies and modes, the characteristic parameters of the gate chamber structure are obtained. Wester-Gard additional mass method is used to simulate structural effects. The first five natural frequencies and periods of the lowering gate chamber structure under normal water level are shown in Table 2. From Table 2, it can be seen that the natural fundamental frequency of gate chamber structure under normal impounding condition is 4.038 Hz, and the second-order natural vibration frequency is similar to the basic frequency. This is mainly due to the independence of each hole in the gate chamber structure and the hoist room on the gate pier. The first and second vibration modes of the gate chamber structure are two independent hoist rooms along the river direction.

**Figure 4:**
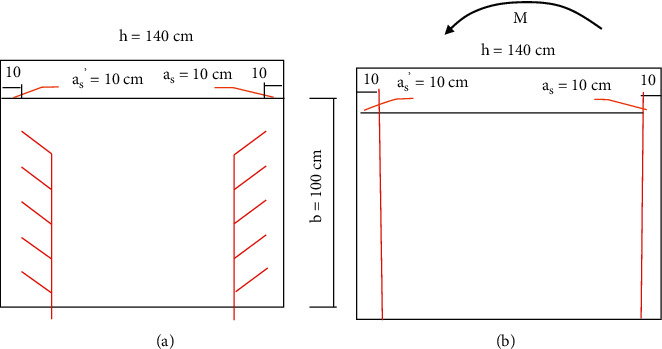
Reinforcement diagram of side pier per unit length of sluice. (a) Cross section. (b) Longitudinal section.

Figure 4 shows the schematic diagram of the side pier and reinforcement of the maximum diameter section of each unit of the sluice. Through the tensile stress review method introduced in Chapter 2.2, the maximum pressure on the longitudinal section of the side pier of each unit can be calculated by equations (1) to (3). The bending moment is 2879.6 kn. M. It can be seen from formula (4) that the maximum allowable tensile stress at the intersection of side pier and gate bottom is 4.41 MPa, equal to 2.00 MPa, which is in line with general safety regulations.

In the stage of sluice modal analysis, according to the calculated diversion water level standard, the upstream water level of the sluice house section is 37.40 m, while the downstream water level of the sluice house section is only 37.40 m. The soil boundary is generally fixed, and the following design modes are generally adopted: (1) fixed boundary + no mass foundation model, (2) fixed boundary + massless foundation model + hydrodynamic pressure, (3) stable boundary + mass base model, and (4) stable boundary + mass basic model + dynamic water pressure; the first 10 natural vibration frequencies of the sluice design are shown in Table 3.

**Figure 5:**
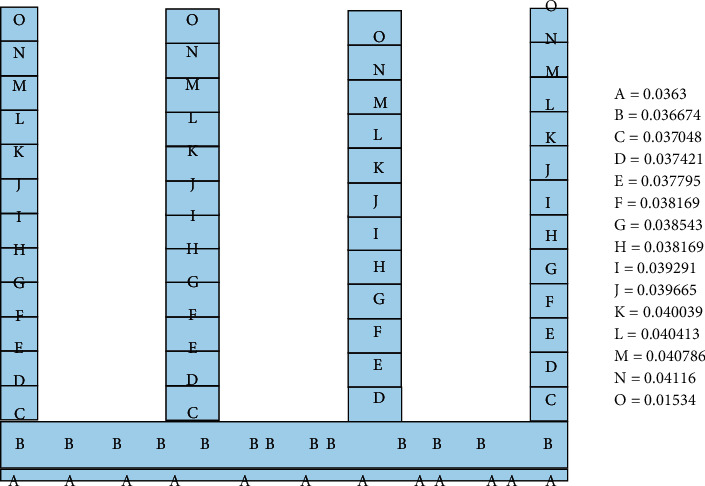
Displacement *Sx* contour map (*m*) of cross section (*z* = 6.64 m) during the Henghe earthquake.

According to the above calculation model and parameters, the dynamic response results of the sluice under the two working conditions are calculated. Now only the displacement diagram of the *X*-direction of the cross section of the sluice chamber structure, frame bridge, and hoisting machine room under the action of the Henghe-direction earthquake is given.  Figure 5 is a displacement diagram in the *X* direction of the cross section (*z* = 0.715 m, *z* = −6.64 m) of the sluice chamber structure under the action of the Henghe earthquake.

The analysis of the displacement results of the sluice structure under the earthquake conditions in the Yokogawa direction shows that the displacement of the bottom plate and the middle part of the top plate of the sluice chamber structure behind the sluice parapet is larger than the displacement on both sides.

Under the two seismic conditions, the maximum displacement of the sluice structure in the *X*, *Y*, and *Z* directions and the deformation of the different structures of the sluice in the *X*, *Y*, and *Z* directions are shown in Table 4.

The analysis of the structural deformation results of the two seismic conditions shows that when the overall model of the sluice including the frame bridge of the sluice and the hoisting machine room is established, the sluice chamber is under the pressure of the side fill in the direction of the river; in the direction of the river, the sluice chamber is constrained horizontally. Under the action of the river-direction earthquake, the sluice structure has a translational displacement in the transverse direction of the river, and its value is 3.61 cm. Under the action of the river-direction earthquake, the sluice chamber structure has no translational displacement.

Table 5 shows the calculation results of stability against sliding of gate chamber structure superimposed by dynamic and static state. It can be seen from Table 3 that fixed boundary is used for soil boundary and mass-free foundation model is used for gate modal analysis. Based on the integration of self-vibration frequency of gate structure and soil, the hydrodynamic pressure of gate chamber also acts on the pier surface through mass unit MASS21. According to the finite element dynamic calculation, the maximum horizontal seismic pressure in the side hole gate house is about 2863.72 kn. Since the earthquake action is random reciprocating, when the ground plane seismic inertial motion faces the downstream, according to the static action calculation, the anti-skid stability safety factor *K* of the gate house structure is 2.30, which meets the requirements. When the ground inertial motion faces the upstream horizontal earthquake, the anti-skid stability safety factor *K* of the gate house structure is 4.64 according to the static action calculation, which meets the requirements.

**Figure 6:**
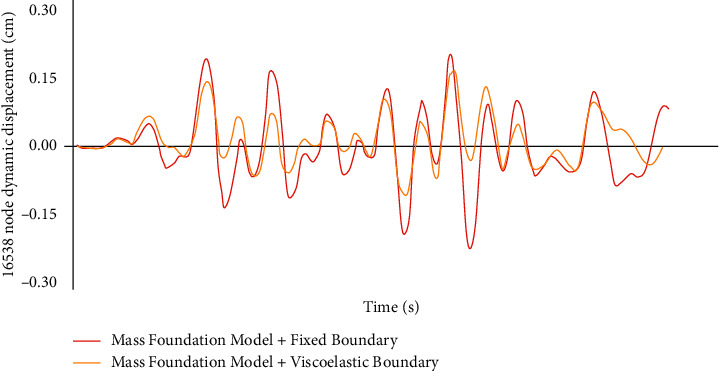
Impact curve of lower boundary form on results of quality basis model.

Because of the dynamic displacement response curve in the Hengchuan direction, the dynamic displacement difference of the typical displacement node between the rack bridge and the hoist room with time is relatively small, and only the dynamic displacement strain curve of the typical node of the gate house with time in the Hengchuan direction is given (see Figure 6.

The comparison results of sluice structure displacement of different models show that the dynamic displacement of the sluice chamber structure is larger than that of the sluice frame bridge and the hoisting machine room. Under the same boundary foundation model, the dynamic displacement of the sluice chamber structure can be calculated: (1) the peak dynamic displacement of the model is smaller than the peak value of the model, (2) the dynamic displacement of the model and the sluice chamber structure, (3) the model is small, and (4) the model is dynamic. In addition, under the same boundary foundation model, the dynamic displacement of the sluice bridge and the hoist room structure is the same due to different time, while the dynamic displacement of the sluice chamber structure is the same at the beginning time. After 1.25 s, the dynamic displacement peak value of the model is higher than that of the model without mass.

## 5. Conclusion

This paper summarizes the damage caused by the earthquake to the gate structure and studies the causes of the damage caused by the earthquake to the gate structure. On the basis of this analysis and research, the basic concept of the seismic analysis and research is systematically expounded; the role of the gate, foundation, and bottom plate in the seismic design is comprehensively investigated; and the whole gate design is calculated and analyzed. Through the finite element numerical simulation technology, the vibration resistance test of the gate house structure is carried out, and the following results are obtained according to the calculation: (1) under the normal horizontal condition, the fundamental frequency of the gate house structure is 3.57 Hz, and the first mode is the vibration of the lifting chamber along the river; (2) when the gate pier intersects with the gate bottom plate, the gate mouth has a tensile stress of 2.0 MPa. According to the quantity of reinforcement here, the maximum tensile stress that the place can bear is about 4.41 MPa, which has met the safety requirements. This value far exceeds the standard value of dynamic axial tensile strength of concrete. The reinforced concrete at the bottom of the bridge falls off, the tendon is exposed, and the crack is expanded. (3) Under normal operation, the maximum sliding stability safety factor of the gate chamber structure is 2.30, which is in line with the safety regulations. According to the guidelines for safety evaluation of sluices, the seismic safety of sluices conforms to the relevant provisions of the standard for seismic design of water conservancy buildings and has design defects that do not affect the overall safety, and its seismic grade is *B*.

## Figures and Tables

**Table 1:** Concrete material parameters.

Material number	Material name	Density	Elastic mold	Poisson's ratio	Standard value of dynamic axial compressive strength	Standard value of dynamic axial tensilestrength
1	Brake floor	2548.00	33.23	0.167	19.66	1.97
2	Pier	2548.00	31.20	0.167	16.22	1.62
3	Highway bridge	2548.00	31.52	0.167	16.72	1.67
4	Open and close the machine room	2548.00	31.52	0.167	16.72	1.67

**Table 2:** Period table of natural frequency of gate chamber structure under different conditions.

Order	Normal water level	
	Frequency	Cycle
1	4.038	0.248
2	4.178	0.239
3	7.708	0.130
4	13.013	0.077
5	13.820	0.072

**Table 3:** First 10 natural vibration frequencies of four models of the sluice.

Order	1/Hz	2/Hz	3/Hz	4/Hz
1	1.720	0.084	0.942	0.084
2	1.932	0.109	0.992	0.109
3	3.606	0.136	1.081	0.136
4	3.686	1.361	1.220	0.961
5	4.302	3.606	1.569	1.059
6	5.885	5.781	1.569	1.099
7	7.039	7.039	1.751	1.569
8	12.180	7.961	1.828	1.569
9	14.584	11.083	1.881	1.748
10	15.482	11.697	1.956	1.764

**Table 4:** Maximum displacement of sluice structures in *X*, *Y*, and *Z* directions under two seismic conditions.

Working condition	*S *_*x*_	*S *_*y*_	*S *_*z*_
Working condition 1	8.87	1.44	0.38
Working condition 2	0.05	0.29	5.24

**Table 5:** Calculation and analysis table of anti-sliding stability of gate chamber structure superimposed by dynamic and static state.

Working condition	Vertical load under static condition	Horizontal load under static condition	Horizontal seismic inertia	Friction coefficient	Anti-skid stability factor	Canonical value
Horizontal seismic inertial force upstream	25197.52	964.93	−2863.72	0.35	4.64	1.10

Horizontal seismic inertial force downstream	25197.52	964.93	2863.72	0.35	2.30	1.10

## Data Availability

The labeled data set used to support the findings of this study can be obtained from the corresponding author upon request.
